# Structures of the promoter-bound respiratory syncytial virus polymerase

**DOI:** 10.1038/s41586-023-06867-y

**Published:** 2023-12-20

**Authors:** Dongdong Cao, Yunrong Gao, Zhenhang Chen, Inesh Gooneratne, Claire Roesler, Cristopher Mera, Paul D’Cunha, Anna Antonova, Deepak Katta, Sarah Romanelli, Qi Wang, Samantha Rice, Wesley Lemons, Anita Ramanathan, Bo Liang

**Affiliations:** Department of Biochemistry, Emory University School of Medicine, Atlanta, GA USA

**Keywords:** Cryoelectron microscopy, Virus structures

## Abstract

The respiratory syncytial virus (RSV) polymerase is a multifunctional RNA-dependent RNA polymerase composed of the large (L) protein and the phosphoprotein (P). It transcribes the RNA genome into ten viral mRNAs and replicates full-length viral genomic and antigenomic RNAs^1^. The RSV polymerase initiates RNA synthesis by binding to the conserved 3′-terminal RNA promoters of the genome or antigenome^2^. However, the lack of a structure of the RSV polymerase bound to the RNA promoter has impeded the mechanistic understanding of RSV RNA synthesis. Here we report cryogenic electron microscopy structures of the RSV polymerase bound to its genomic and antigenomic viral RNA promoters, representing two of the first structures of an RNA-dependent RNA polymerase in complex with its RNA promoters in non-segmented negative-sense RNA viruses. The overall structures of the promoter-bound RSV polymerases are similar to that of the unbound (apo) polymerase. Our structures illustrate the interactions between the RSV polymerase and the RNA promoters and provide the structural basis for the initiation of RNA synthesis at positions 1 and 3 of the RSV promoters. These structures offer a deeper understanding of the pre-initiation state of the RSV polymerase and could aid in antiviral research against RSV.

## Abstract (web summary)

A study reports cryogenic electron microscopy structures of the respiratory syncytial virus polymerase bound to its genomic and antigenomic viral RNA promoters.

## Main

**Fig. 1:**
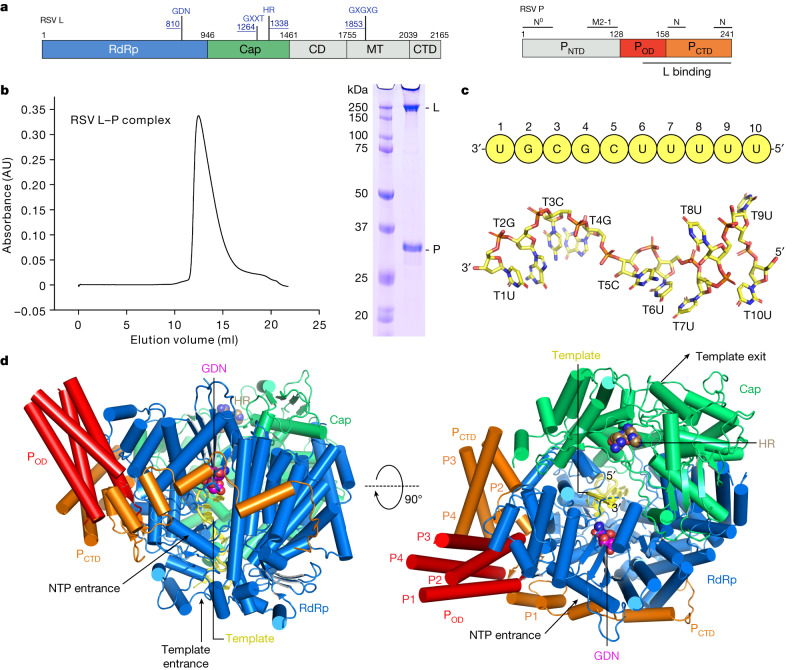
Cryo-EM structure of the RSV polymerase in complex with the leader (Le10) promoter of the RSV genome. **a**, Schematic representation of the RSV polymerase (L–P complex) with labelled domain boundaries and P regions interacting with N^0^ (RNA-free N), M2-1, N (RNA-bound N) and L proteins. The domains with missing density are coloured grey. The key residues for each functional domain are highlighted above with blue and underlined residue numbers. AU, absorbance units. **b**, The size-exclusion chromatography and SDS–polyacrylamide gel electrophoresis (PAGE) gel results show the quality and purity of the purified RSV L–P complex. All data shown are representative of three independent experiments (*n* = 3), and the uncropped SDS–PAGE image is shown in Supplementary Fig. 1. **c**, The sequence and structure of the Le10 RNA template in the Le10-bound RSV polymerase. The residues are labelled as the template (T) position number and base name. **d**, Schematic diagrams of the RSV polymerase in complex with Le10 in two orientations. The atomic model of the RdRp domain (blue) and the Cap domain (green) of RSV L, as well as P_OD_ (red) and P_CTD_ (orange) of a tetramer of RSV P, are shown. The P_OD_ and P_CTD_ domains of each P monomer are labelled as P1, P2, P3 and P4. The missing domains (coloured grey) include the CD, the MT domain and the CTD of RSV L, and the P_NTD_ of RSV P. The domains are colour-coded, as in **a**, and the Le10 RNA template is coloured yellow, as in **c**. The ‘GDN’ motif (residues 810–812) from the RdRp domain is shown as magenta spheres, and the ‘HR’ motif (residues 1338–1339) from the Cap domain is shown as light brown spheres.

RSV is a highly contagious human pathogen that causes severe respiratory tract infections, particularly in young children, older adults and individuals who are immunocompromised^3,4^. RSV is responsible for 55% of infant infections, making it the second deadliest infectious agent in children under 1 year of age^3,4^. RSV belongs to the Pneumoviridae family in the order of Mononegavirales, also known as non-segmented negative-sense (NNS) RNA viruses. The RSV RNA genome consists of a single-strand negative-sense RNA encapsidated by the nucleoprotein (N) to form a ribonucleoprotein particle. This ribonucleoprotein particle is transcribed and replicated by the RSV RNA-dependent RNA polymerase (RdRP), which is composed of a multifunctional large (L) polymerase protein and a cofactor phosphoprotein (P). The RSV L protein consists of five domains: the RdRp domain, the capping domain (Cap), the connector domain (CD), the methyltransferase domain (MT) and the carboxy-terminal domain (CTD). Of these, three domains—RdRp, Cap and MT—possess enzymatic activity and are responsible for RNA synthesis. The RSV P protein, by contrast, has three domains: the amino-terminal domain (P_NTD_), the oligomerization domain (P_OD_) and the CTD (P_CTD_;) (Fig. 1a).

In recent years, cryogenic electron microscopy (cryo-EM) has been used to obtain several structures of the apo polymerases from different NNS RNA viruses. These structures have provided critical insights into the enzymatic domains responsible for RNA synthesis (reviewed in refs. ^5,6^). These cryo-EM structures include those from vesicular stomatitis virus (VSV), rabies virus (RABV), RSV, human metapneumovirus (HMPV), parainfluenza virus (PIV), Newcastle disease virus (NDV) and Ebola virus (EBOV)^7–16^. These structures share strikingly similar architectures, with only the RdRp and Cap domains being consistently modelled among the five domains of the L protein. Notably, the other three domains (CD, MT and CTD) of the L protein are missing in the cryo-EM structures of RSV and HMPV (both from the Pneumoviridae family), as well as in that of EBOV (from the Filoviridae family). However, these domains can be modelled in VSV and RABV (both from the Rhabdoviridae family), as well as PIV and NDV (both from the Paramyxoviridae family; reviewed in refs. ^5,6^).

The RSV polymerase recognizes the promoter sequences at the 3′ ends of the genome and antigenome, namely the leader (Le) and trailer complementary (TrC) sequences, respectively^1,17–23^. These sequences are highly similar, differing by only 1 nucleotide (nt) in the first 11 nt^1,17,18^. One unique feature of RSV RNA synthesis is that the RSV polymerase can initiate RNA synthesis at two distinct positions: positions 1 and 3 of the promoters^19–23^. Specifically, the RSV polymerase initiates the replication of genome or antigenome RNA at position 1 of Le or TrC promoters, respectively, but initiates mRNA transcription at position 3 of the Le promoter in cell-based assays^19^. Notably, in vitro experiments show that the RSV polymerase can de novo synthesize RNA at positions 1 and 3 using short Le and TrC templates^19,20,24,25^. Despite extensive studies, the structural basis for the two distinct initiation positions remains unclear, mainly owing to the lack of atomic-resolution structures of polymerases complexed with RNA promoters.

We report here cryo-EM structures of the RSV polymerase complexed with RNA templates derived from 3′ genomic and antigenomic RNA promoters, representing two of the first structures of an NNS viral RdRP in complex with its RNA promoters. We used the high-quality recombinant RSV polymerase (L–P complex) and an in vitro transcription assay to identify suitable RNA templates for structural analysis^10,24,25^. The overall structures of the promoter-bound polymerases are similar to that of the apo polymerase. RSV L, but not RSV P, directly interacts with the RNA templates. Our structures uncovered that the RSV L interfaces with the conserved 3′ Le or TrC promoter sequences, corresponding to the initiation at positions 3 and 1, respectively. Our structures showed that the ‘supporting helix’ (residues 666–676) that was missing in the apo polymerase^10,11^ could be modelled in both Le and TrC promoter-bound RSV polymerase complexes. We found that the ‘supporting loop’ (residues 656–665) stabilizes the first nucleotide of the Le promoter and provided structural evidence that the RSV polymerase prefers a guanine (G) base at the +1 catalytic site for RNA synthesis initiation. In summary, this study provides valuable insights into the structural basis for RSV RNA synthesis initiation, which may facilitate the development of new antiviral drugs and vaccines.

## Structure determination

Large quantities of homogeneous full-length wild-type L and P proteins were co-expressed in insect cells and co-purified through affinity, ion-exchange and size-exclusion chromatography, as previously described^10^ (Fig. 1b). The purified recombinant RSV polymerase (L–P complex) was active in de novo and primer-based transcription assays using short Le or TrC promoter sequences, although non-processive in the absence of the N protein^24,25^. We determined that the minimal template length required for de novo RSV RNA synthesis is 8 nt, whereas templates of 10 nt or longer show similar functional activities^24^. To analyse the structures, we selected 10-nt-long RNA oligonucleotides from the 3′ end of Le and TrC promoters and named them Le10 (3′-UGCGCUUUUU) and TrC10 (3′-UGCUCUUUUU) RNA templates. We assembled the RSV polymerase in complex with Le10 (Le10-bound) or TrC10 (TrC10-bound) by incubating the RSV polymerase with the respective RNA promoters.

**Extended Data Fig. 1:**
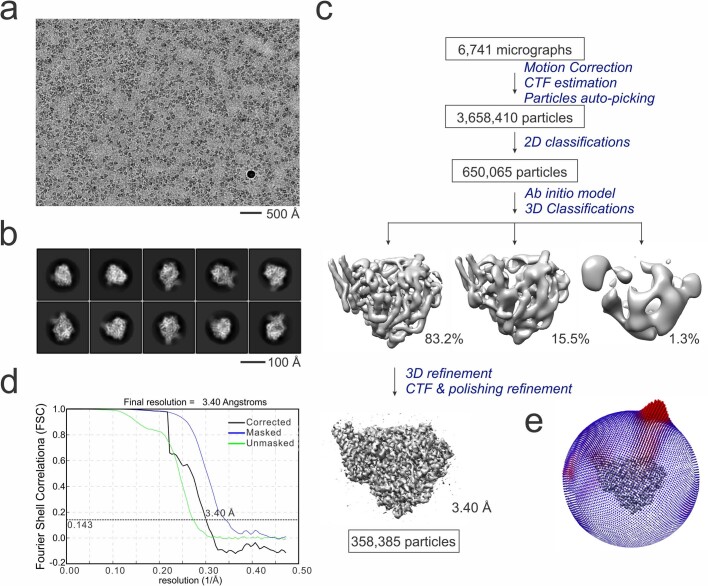
Cryo-EM data processing of the respiratory syncytial virus (RSV) polymerase in complex with leader promoter Le10. (**a**) The representative micrograph (1 out of 6,741 micrographs) of the RSV polymerase (L:P complex) in complex with leader promoter Le10. Scale bar: 500 Å. (**b**) The representative 2D class averages obtained from the reference-free 2D classification. Scale bar: 100 Å. (**c**) The datasets, classification, and refinement procedures used in this complex. (**d**) The gold-standard Fourier shell correlation (FSC) curves for cross-validation between the corrected, masked, and unmasked maps. (**e**) The angular distribution heatmap of particles used for refinement.

**Extended Data Fig. 2:**
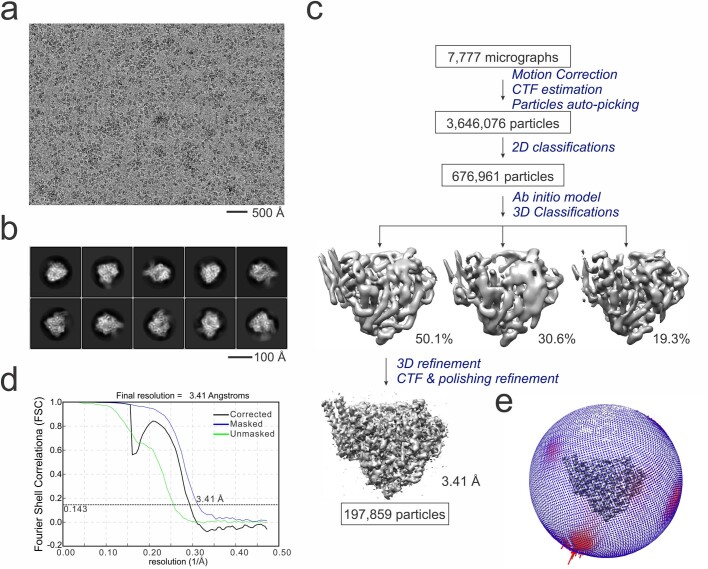
Cryo-EM data processing of RSV polymerase in complex with trailer complementary promoter TrC10. (**a**) The representative micrograph (1 out of 7,777 micrographs) of the RSV polymerase (L:P complex) in complex with trailer complementary promoter TrC10. Scale bar: 500 Å. (**b**) The representative 2D class averages obtained from the reference-free 2D classification. Scale bar: 100 Å. (**c**) The datasets, classification, and refinement procedures used in this complex. (**d**) The gold-standard Fourier shell correlation (FSC) curves for cross-validation between the corrected, masked, and unmasked maps. (**e**) The angular distribution heatmap of particles used for refinement.

**Extended Data Fig. 3:**
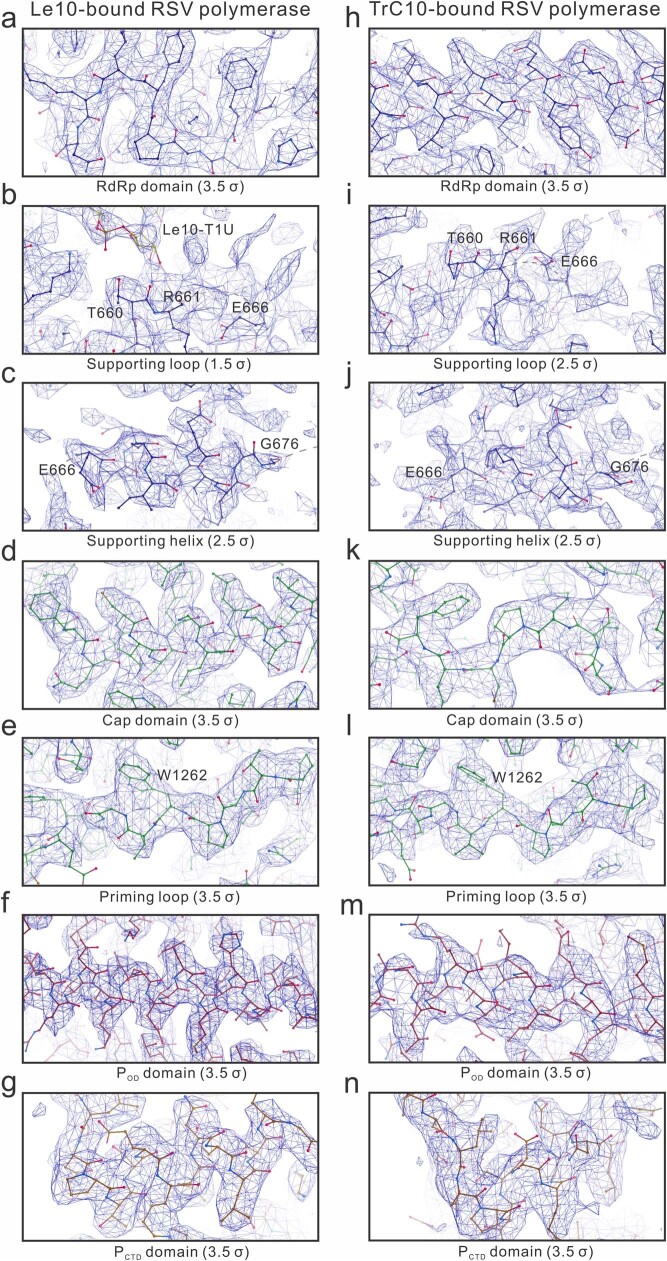
The representative models fit with electron microscopy densities in Le10 and TrC10-bound RSV polymerase complexes. Electron microscopy density segments for representative regions of the Le10 (**a**-**g**) and TrC10 (**h**-**n**) bound RSV polymerase complexes. The models of RdRp domain (**a**, **h**), supporting loop (**b**, **i**), supporting helix (**c**, **j**), Cap domain (**d**, **k**), priming loop (**e**, **l**), P_OD_ (**f**, **m**), and P_CTD_ (**g**, **n**), fitting with the density maps at the counter level of 1.5 σ, 2.5 σ or 3.5 σ, were shown with the same color scheme as Fig. 1.

We collected cryo-EM micrographs of the Le10-bound and TrC10-bound RSV polymerases using several 300-kV Thermo Fisher Scientific Titan Krios microscopes and Gatan K3 cameras. We processed and analysed the collected data using RELION v3.1.3, which included two-dimensional (2D) and 3D classification, refinement and polishing^26^. We picked 3,658,410 particles of the Le10-bound RSV polymerase and 3,646,076 particles of the TrC10-bound RSV polymerase using crYOLO^27^. Further data processing and refinement resulted in 3D reconstructions of 3.40 Å and 3.41 Å resolution for the Le10-bound and TrC10-bound RSV polymerase, respectively (Extended Data Figs. 1 and 2 and Supplementary Table 1). We built the atomic models of the Le10-bound and TrC10-bound RSV polymerase structures using UCSF ChimeraX and COOT^28,29^. We used the apo RSV polymerase (Protein Data Bank (PDB) ID: 6UEN) as the initial model for model building. We refined the atomic models using PHENIX and COOT software, and model geometries were validated using MolProbity^29–31^. Illustrations depicting how the representative models of the priming loop, supporting loop and supporting helix, as well as the RdRp, Cap, P_OD_ and P_CTD_ domains, fitted with the cryo-EM maps in the Le10-bound and TrC10-bound RSV polymerase complexes, are shown in Extended Data Fig. 3. Finally, we prepared the figures using PyMol^32^.

We first discuss the Le10-bound RSV polymerase structure for clarity and simplicity, followed by the TrC10-bound RSV polymerase structure.

## Overview of Le10-bound RSV polymerase

The cryo-EM structure of the Le10-bound RSV polymerase revealed the presence of the RdRp and Cap domains of the L protein and the P_OD_ and P_CTD_ domains of the P protein. Conversely, the CD, MT and CTD domains of the L protein and the P_NTD_ are disordered and not visible, consistent with the apo polymerase structure^10,11^ (Fig. 1a,d). The RdRp and Cap domains of the RSV L form a ‘bowl’ structure that provides sufficient space to accommodate the RNA template and the newly synthesized RNA product^10^ with a buried surface area between the RdRp and Cap of 4,666  Å^2^. The ‘catalytic pocket’ for RNA synthesis is located inside the bowl with the catalytic ‘GDN’ motif (residues 810–812) from the RdRp domain and the catalytic ‘HR’ motif (residues 1338–1339) from the Cap domain situated at opposite locations. The tetrameric RSV P proteins dock to the RdRp domain with a buried interface area of about 4,500 Å^2^ with one P_CTD_ wrapping around the NTP entrance channel outside the centre of the RdRp domain near the ‘GDN’ motif (Fig. 1d).

**Fig. 2:**
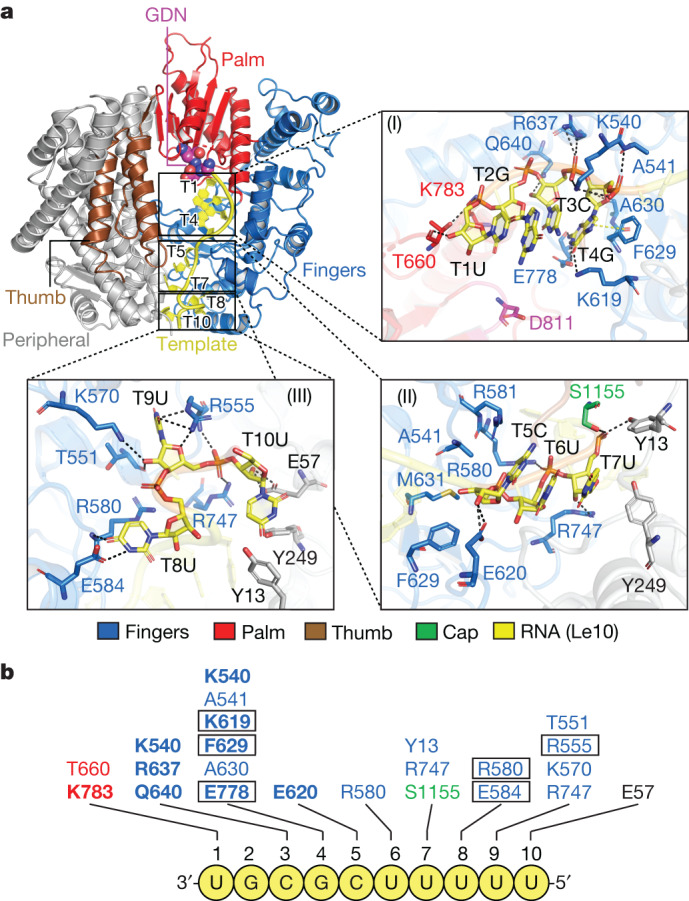
Structural basis of the interactions between RSV polymerase (L–P) and RNA template Le10. **a**, The interactions between the RSV L protein and Le10 RNA template can be divided into three parts: (I) T1–T4, (II) T5–T7 and (III) T8–T10. The zoomed-in insets show the detailed interactions of RSV L residues and three parts of RNA with dashed lines. The conventional right-hand ‘fingers–palm–thumb’ polymerase fold of the RdRp domain of RSV L is shown as ‘fingers’ (blue), ‘palm’ (red) and ‘thumb’ (brown). The Cap domain is coloured green, and the peripheral regions are coloured grey. The Le10 RNA is coloured yellow. The ‘GDN’ motif is shown as magenta spheres. **b**, Residues of RSV L involved in the interactions with Le10. The residues are coloured the same as in **a**. Residues in rectangles interact with RNA bases. Residues in bold are conserved across NNS RNA viruses.

**Extended Data Fig. 4:**
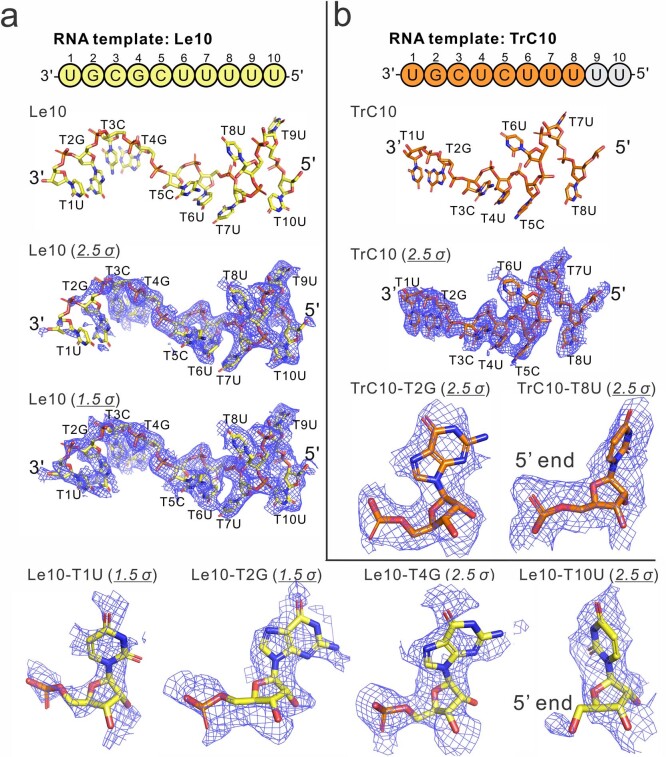
The sequences, models, and cryo-EM densities of the RNA templates. (**a**) The sequences, models, and cryo-EM densities of leader promoter Le10 (the position 3 pre-initiation state). The electron density maps of Le10 were shown at the contour levels of 2.5 and 1.5 σ. Detailed electron density maps for Le10-T1U (1.5 σ), Le10-T2G (1.5 σ), Le10-T4G (2.5 σ), and Le10-T10U (2.5 σ) were also shown. (**b**) The sequences, models, and cryo-EM densities of trailer complementary promoter TrC10 (the position 1 pre-initiation state.) The electron density map of TrC10 was shown at the contour levels of 2.5 σ. Detailed electron density maps for TrC10-T2G (2.5 σ) and TrC10-T8U (2.5 σ) were also shown. The residue is denoted by the template (T) position number with the base name. Note that the 5'ends of the visible RNA residues Le10-T10U (the ending hydroxyl group) and TrC10-T8U (the internal phosphate group) are different, confirming the difference between the modelings of Le10 and TrC10.

In the Le10-bound RSV polymerase structure, the 3′ end of an incoming Le10 RNA template enters the template entrance channel, briefly touches the edge of the Cap domain, and runs towards the ‘GDN’ motif of the catalytic pocket from the bottom of the bowl. There are 6 nt in the template entrance channel and 4 nt in the catalytic pocket (Figs. 1c and 2a and Extended Data Fig. 4a).

For clarity, we labelled each nucleotide in the Le10 RNA sequence (3′-UGCGCUUUUU) with a numerical position, starting from the 3′ end and naming the template (T) with position number and base name (for example, T1U). The second (T2G) and fourth (T4G) positions in Le10 RNA are larger purine bases (G), whereas the other positions are smaller pyrimidine bases (U or C). The 3D map of the Le10-bound RSV polymerase enabled us to model T1U and T2G at the counter level of 1.5*σ* and T3C to T10U at the counter level of 2.5*σ* (Extended Data Fig. 4a). The sufficient resolution of the 3D map allowed us to determine that T2G and T4G correspond to the larger purine base G. The map of the other positions is consistent with smaller pyrimidine bases (Extended Data Fig. 4a). Although GTP and CpNpp were supplemented, no cryo-EM density was observed for the RNA product. Additionally, the ‘priming loop’ (residues 1250–1280) of the Cap domain was distant from the active site, indicating that the structure represents a pre-initiation state for de novo RNA synthesis at position 3 of the template.

## Interactions between RSV L and Le10

The RdRp domain of the RSV L adopts a conventional right-hand ‘fingers–palm–thumb’ polymerase fold, as illustrated in Fig. 2a. Most of the interaction between Le10 RNA promoter and RSV L occurs at the ‘fingers’ (blue) and ‘peripheral’ (grey) regions, which form a composite tunnel for the RNA template to enter (Fig. 2a). The RNA–protein interactions can be divided into three parts: (I) positions 1–4 of Le10 and the catalytic pocket; (II) positions 5–7 of Le10 and the template entrance channel; (III) positions 8–10 of Le10 and the gate of the template entrance channel (Fig. 2a(I–III)).

**Extended Data Fig. 5:**
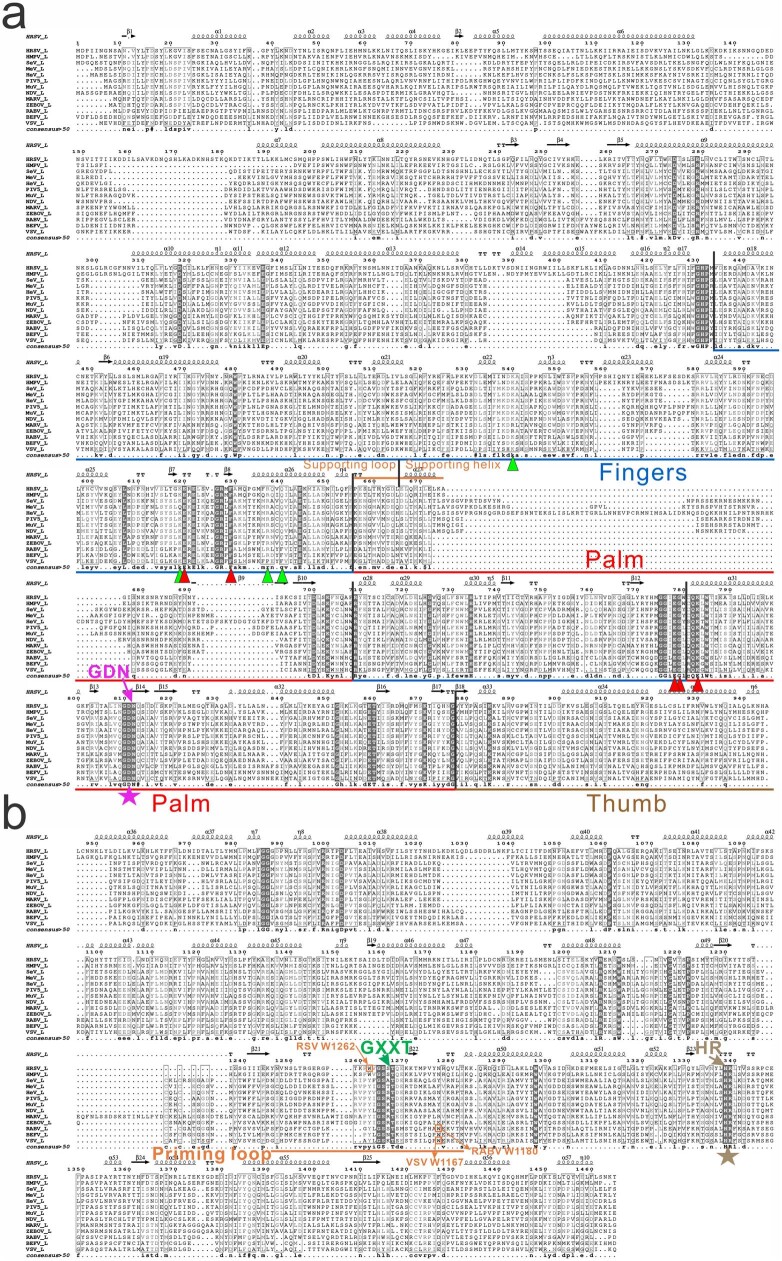
Sequence alignment of the RdRp and Cap domains among nonsegmented negative-sense (NNS) RNA viruses. (**a**) Sequence alignment of the RdRp domain of the L proteins among NNS RNA viruses. The RdRp domain features a conventional right-hand ‘fingers-palm-thumb’ polymerase fold. The ‘fingers’, ‘palm’, and ‘thumb’ regions are colored blue, red, and brown, respectively. The supporting loop and helix were colored orange. The catalytic ‘GDN’ motif is labeled with a magenta star. Highly conserved and identical (100% conservation) residues involved in the interaction with the RNA template are labeled with green and red triangles, respectively. (**b**) Sequence alignment of the Cap domain of the L proteins among NNS RNA viruses. The catalytic ‘HR’ motif is labeled with a light brown star. The ‘GXXT’ motif is highlighted and colored green. The priming loop region was colored orange with potential priming residues in RSV (W1262), RABV (W1180), and VSV (W1167) highlighted. The secondary structures of the HRSV L identified are shown above the alignment. The alignment graphics were prepared with Multalin and ESPript. The sequences of the L proteins here were obtained from the UniProt website: HRSV, human respiratory syncytial virus (https://www.uniprot.org/uniprotkb/P28887/entry); HMPV, Human metapneumovirus (https://www.uniprot.org/uniprotkb/Q6WB93/entry); SeV, Sendai virus (https://www.uniprot.org/uniprotkb/O55528/entry); MeV, Measles virus (https://www.uniprot.org/uniprotkb/P12576/entry); HeV, Hendra virus (https://www.uniprot.org/uniprotkb/O89344/entry); PIV5, Parainfluenza virus 5 (https://www.uniprot.org/uniprotkb/Q88434/entry); MuV, Mumps virus (https://www.uniprot.org/uniprotkb/P30929/entry); NDV, Newcastle disease virus (https://www.uniprot.org/uniprotkb/Q9DLD3/entry); MARV, Lake Victoria marburgvirus (https://www.uniprot.org/uniprotkb/P31352/entry); ZEBOV, Zaire ebolavirus (https://www.uniprot.org/uniprotkb/Q05318/entry); RABV, Rabies virus (https://www.uniprot.org/uniprotkb/P11213/entry); BEFV, Bovine ephemeral fever virus (https://www.uniprot.org/uniprotkb/Q9E784/entry); VSV, Vesicular stomatitis Indiana virus (https://www.uniprot.org/uniprotkb/P03523/entry).

Specifically, the T1U backbone of Le10 interacts with the residue T660 from the supporting loop and K783 of the ‘palm’, contributing to the RNA template stability. No interaction was found between T2G and RSV L. Positions 3 and 4 of Le10 (T3C and T4G) are opposite to the catalytic residue D811, which occupies the −1 and +1 catalytic sites for RNA synthesis. The backbone of T3C interacts with residues K540, R637 and Q640 from the fingers region to stabilize the RNA template during initiation. T4G is opposite to the incoming NTP in the +1 catalytic site. The base of T4G interacts with residues K619, F629 and E778, all of which are conserved across the polymerases of NNS RNA viruses (Extended Data Fig. 5). The oxygen (O6) of the T4G base interacts with the nitrogen (NZ) of residue K619, and the nitrogen (N2) of the T4G base interacts with the oxygen (OE1) of residue E778. This may explain why RSV polymerase prefers a G at the +1 catalytic site for de novo RNA synthesis initiation, as G is the only base that contains an O atom towards K619 and an N atom towards E778. The T4G base forms π–π interactions with residue F629 (Fig. 2a(I),b). The bases of positions 5 to 7 of Le10 are stacked with each other (Fig. 2a(II)). The RSV L interacts with the backbone but not the bases of positions 5–7 of Le10 through residues E620, R580, Y13, R747 and S1155 (Fig. 2a(II),b). Note that residue S1155 is from the Cap domain. Positions 8 and 9 of Le10 (T8U and T9U) are in two small pockets formed by the fingers region and part of the Cap domain, which involve residues R580 and E584 for T8U and T551, R555, K570 and R747 for T9U. The base of T8U interacts with residues R580 and E584, and the base of T9U interacts with residue R555. T10U is located at the gate of the template entrance channel and its backbone interacts with the residue E57 (Fig. 2a(III),b).

## TrC10-bound RSV polymerase

**Fig. 3:**
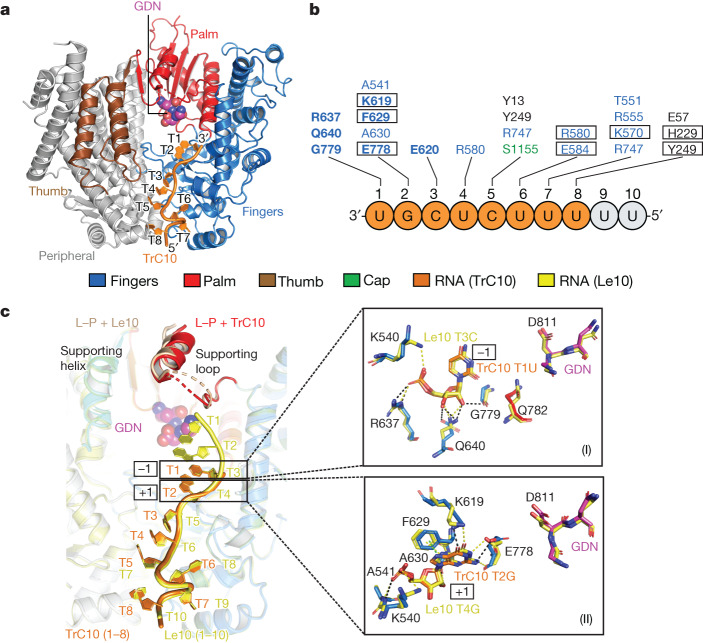
Cryo-EM structure of the RSV polymerase in complex with the trailer complementary (TrC10) promoter of the RSV antigenome. **a**, The interactions between the RdRp domain of RSV L and TrC10 RNA template. The RdRp domain is coloured the same as in Fig. 2a, and the TrC10 is coloured orange. **b**, Residues of the RSV L involved in the interactions with TrC10. The residues are coloured the same as in **a**. The RNA residues with missing density are coloured grey. Residues in rectangles interact with RNA bases. Residues in bold are conserved across NNS RNA viruses. **c**, The superimposition of the RSV polymerase in complex with Le10 (Le10-bound) and TrC10 (TrC10-bound). The Le10 RNA is shown in yellow, as in Fig. 2a, and the TrC10 RNA is shown in orange, as in **a**. The interactions of Le10 T3C and TrC10 T1U (at the −1 catalytic site) and Le10 T4G and TrC10 T2G (at the +1 catalytic site) with RSV polymerase (L–P) are shown in the right panels (I) and (II), respectively. The ‘GDN’ motif is shown as magenta spheres. The supporting helix (residues 666–676) and supporting loop (residues 656–665) are shown for Le10-bound (tan) and TrC10-bound (red) RSV polymerases. Note that part of the supporting loop is too flexible to build in both structures.

The sequence of TrC10 is similar to that of Le10, except for the base composition at position 4: Le10 has a purine base G, whereas TrC10 has a pyrimidine base U (Extended Data Fig. 4b). We were able to identify the larger base of TrC10 T2G at the counter level of 2.5*σ*. Moreover, the electron density of the 5′-end RNA residue TrC10 T8U contains a phosphate group, which is different from that of Le10 T10U (a hydroxyl group, ‘–OH’), confirming that the RNA template conformations in Le10- and TrC10-bound structures are different (Extended Data Fig. 4). As a result, in the TrC10-bound RSV polymerase complex, we modelled the first 8 nt from the 3′ end of TrC10 RNA with the first 2 nt (T1U and T2G) in the catalytic pocket and the other 6 nt (T3C to T8U) in the template entrance channel (Fig. 3a). The T1U backbone of TrC10 interacts with residues R637, Q640 and G779 from the fingers region. T2G of TrC10 interacts with residues A541, K619, F629, A630 and E778. Among them, the T2G base of TrC10 has π–π interactions with F629. The polar oxygen and nitrogen atoms of the T2G base interact with residues K619 and E778 (Fig. 3b). The remaining 6 nt of TrC10 are in the template entrance channel and interact with more than a dozen residues, including residues E620, R580, Y13, Y249, R747, S1155, E584, T551, R555, K570, E57, H229 and Y249. The T6U base of TrC10 interacts with residues R580 and E584, and the T7U base of TrC10 interacts with residue K570 (Fig. 3b). These interactions are mostly similar to those observed in positions 5–10 of Le10.

Comparing the TrC10- and Le10-bound RSV polymerase complexes, the visible 8 nt of TrC10 RNA correspond to positions 3–10 of the Le10 RNA (Fig. 3c). Despite the difference in RNA, the supporting helix can be modelled at the counter level of 2.5*σ* in both structures (Extended Data Fig. 3c,j). The loop before the supporting helix is flexible and we can build only part of the supporting loop at the counter level of 1.5*σ*, including the residue T660 that interacts with the backbone of Le10 T1U (Extended Data Fig. 3b). The T1U of TrC10 and T3C of Le10, occupying the catalytic sites −1, are opposite to the ‘GDN’ motif and interact with similar residues from the RdRp domain (Fig. 3c(I)). Both Le10 and TrC10 promoters prefer a G base at the catalytic site +1 for de novo RNA synthesis initiation in the promoter-bound RSV polymerase structures. The G base is stabilized by hydrogen bonds with the side chain of residues K619 and E778 and π–π interactions with residue F629 of the RSV L (Fig. 3c(II)).

## Comparison with apo RSV polymerase

**Extended Data Fig. 6:**
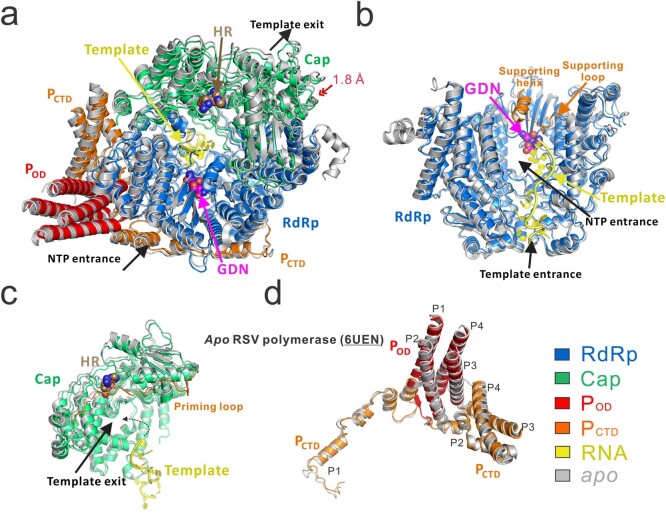
Structural comparison of *apo* and promoter-bound RSV polymerase (L:P). The L and P proteins are almost identical in Le10-bound and TrC10-bound RSV polymerase structures. Thus, only Le10-bound RSV polymerase is used to align with the *apo* RSV polymerase. (**a**) Overall comparison of *apo* (gray) and Le10-bound RSV polymerases (L:P) (colored the same as Fig. 1). Compared with *apo* RSV L, the Cap domain of Le10-bound RSV L shifts inward by approximately 1.8 Å (red double-arrow), yielding a more compact catalytic pocket. (**b**) Structural comparison of the RdRp domains of the L protein between *apo* and Le10-bound RSV polymerases. The supporting helix and the supporting loop are colored orange. (**c**) Structural comparison of the Cap domains of the L protein between *apo* and Le10-bound RSV polymerases. The priming loop is colored orange. (**d**) Structural comparison of the RSV P between *apo* and Le10-bound RSV polymerases (L:P). The ‘GDN’ motif is shown in magenta spheres, and the ‘HR’ motif is shown in light brown spheres.

The overall structures were similar (Extended Data Fig. 6a) when the promoter-bound RSV polymerases were superimposed onto the apo RSV polymerase (PDB ID: 6UEN). However, a notable difference was observed for the supporting helix of the RdRp domain of RSV L. In the absence of RNA, the supporting helix is flexible and not visible in the cryo-EM map of the apo RSV polymerase. By contrast, the promoter-bound structure shows a solid density for the supporting helix, suggesting that the RNA binding stabilizes the conformation of the supporting helix, and the RSV L undergoes conformational changes during different stages of RSV RNA synthesis (Extended Data Figs. 3c,j and 6b).

When fixing the location of the RdRp domain, the RNA binding causes the Cap domain of RSV L to shift inward by approximately 1.8 Å, resulting in a more compact catalytic pocket (Extended Data Fig. 6a, red arrow). This suggests that RNA binding leads to slight conformational changes in RSV L. However, the Cap domain alone does not exhibit notable differences between the apo and promoter-bound structures. The priming loop remains distant from the active site (Extended Data Figs. 3e,l and 6c), indicating that the Cap domain plays a lesser role in template recognition than other domains.

In the apo RSV polymerase, the RSV P is identical to the promoter-bound RSV polymerase, but certain loops connecting the helix in the RSV P show worse density, suggesting greater flexibility following RNA binding (Extended Data Fig. 6d).

## Comparison with other viral RNA polymerases

**Fig. 4:**
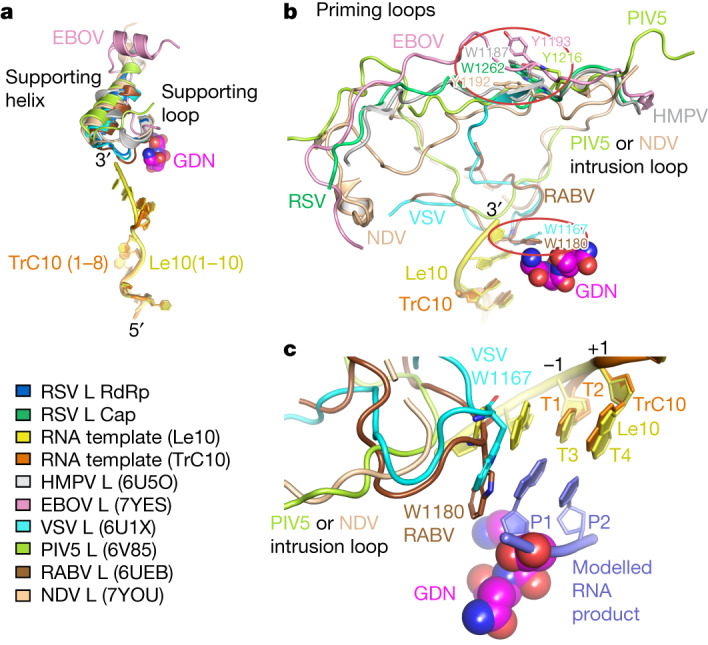
Structural comparison of the RNA polymerases from different NNS RNA viruses. **a**, Structural comparison of the supporting loop and helix in RdRp domains of RNA polymerases from RSV (blue), HMPV (PDB: 6U5O; grey), EBOV (PDB: 7YES; pink), VSV (PDB: 6U1X; cyan), PIV5 (PDB: 6V85; yellow-green), RABV (PDB: 6UEB; brown) and NDV (PDB: 7YOU; tan). **b**, Structural comparison of the priming and intrusion loops in Cap domains of the RNA polymerases from viruses as shown in **a**. The RSV Cap domain is coloured green and the other viruses’ polymerases are coloured the same as **a**. Potential priming residues (W or Y) in priming loops are highlighted with red ovals. **c**, A zoomed-in view of the modelled RSV product (light blue) in the initiation state interacting with modelled priming loops from VSV (PDB: 6U1X; cyan) and RABV (PDB: 6UEB; brown). The intrusion loops of PIV5 (PDB: 6V85; yellow-green) and NDV (PDB: 7YOU; tan) are shown. The ‘GDN’ motif is shown as magenta spheres.

**Extended Data Fig. 7:**
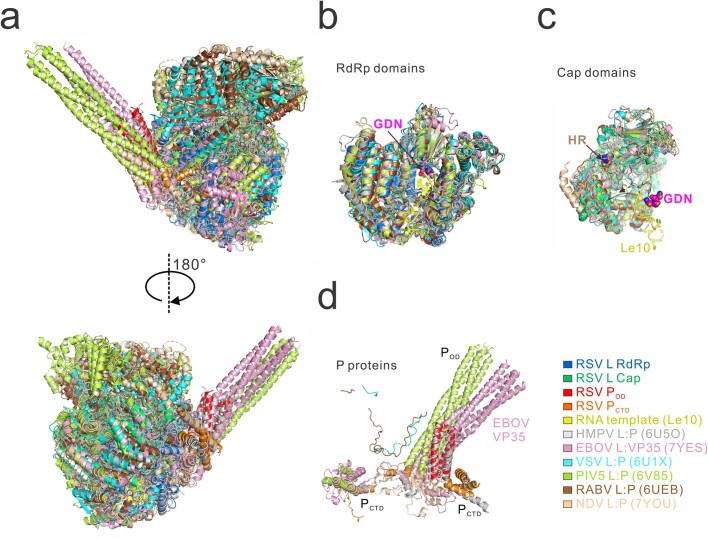
Structural comparison of the RNA polymerases from different NNS RNA viruses. (**a**) The overall structural comparison of the RNA polymerases from RSV (colored as Fig. 1d), HMPV (PDB: 6U5O, gray), EBOV (PDB: 7YES, pink), VSV (PDB: 6U1X, cyan), PIV5 (PDB: 6V85, limon), RABV (PDB: 6UEB, brown) and NDV (PDB: 7YOU, tan). (**b**) Structural comparison of the RdRp domains of RNA polymerases in panel (**a**). (**c**) Structural comparison of the Cap domains of the RNA polymerases in panel (**a**). (**d**) Structural comparison of the P proteins, or VP35 from EBOV, of the RNA polymerases in panel (**a**). The ‘GDN’ motif is shown as magenta spheres, and the ‘HR’ motif is shown as light brown spheres. The RNA template Le10 is colored yellow.

With the recent advancements in cryo-EM technology, the structures of RNA polymerases from several NNS RNA viruses have been determined^7–15^. This study presents two of the first cryo-EM structures of an NNS viral polymerase in complex with its RNA templates. To compare our findings with those for other viruses, we superimposed the RSV polymerase in the promoter-bound RSV polymerase structures with RNA polymerases from other NNS RNA viruses, including HMPV, VSV, RABV, PIV5, EBOV and NDV^7–15^. These polymerases share a similar overall structure. Notably, three domains (CD, MT and CTD) of the L protein can be modelled in VSV, RABV, PIV and NDV, but not in RSV, HMPV or EBOV (Extended Data Fig. 7a). These RNA polymerases share similar structures in the RdRp and Cap domains of L proteins (Extended Data Fig. 7b,c), with notable differences observed in the supporting helix of the RdRp domain (Fig. 4a) and the priming and intrusion loops of the Cap domains (Fig. 4b,c). For instance, in the EBOV polymerase, the supporting helix is more distant with a long supporting loop (Fig. 4a). In VSV and RABV L, the priming loop inserts into the catalytic pocket and remains near the ‘GDN’ motif, whereas in RSV, HMPV, EBOV, NDV and PIV5, this priming loop swings away from the ‘GDN’ motif (Fig. 4b). This difference suggests that they are in different stages of RNA synthesis. In RSV L, the ‘intrusion loop’ (residues 1329–1352), which contains the ‘HR’ motif from the Cap domain, is adjacent to the priming loop and away from the ‘GDN’ motif. Notably, in PIV5 and NDV L proteins, the intrusion loop is close to the ‘GDN’ motif instead of the priming loop (Fig. 4b), which was thought to regulate transcription initiation^13^. In addition, the P proteins from RSV, HMPV, PIV and NDV and VP35 from EBOV are all tetramers and adopt a similar interaction pattern with the L proteins (Extended Data Fig. 7d). However, in VSV and RABV polymerase (L–P), some fragments of P were found to wrap around the CTD domain (Extended Data Fig. 7d).

**Extended Data Fig. 8:**
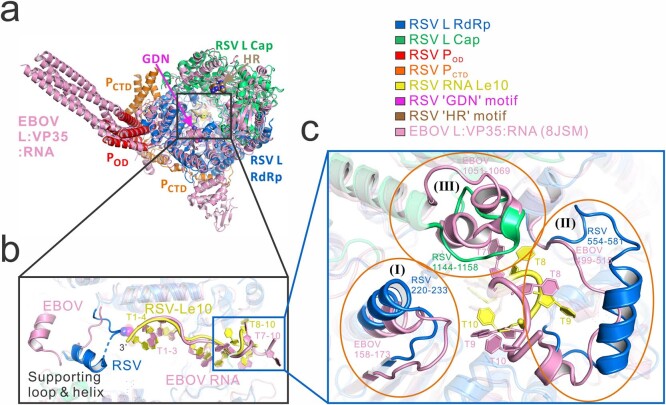
Structural comparison between the viral RNA promoter-bound EBOV polymerase (L-VP35-RNA) complex and Le10-bound RSV polymerase complex. (**a**) The overall structural comparison of the viral RNA promoter-bound EBOV polymerase (L-VP35-RNA) complex (PDB: 8JSM, pink) and Le10-bound RSV polymerase complex (PDB: 8SNX, colored as Fig. 1d). (**b**) Close view of the RNA in RSV and EBOV polymerase complexes. (**c**) Close view of the gate region of the template entrance channel in RSV and EBOV polymerase complexes. The ‘GDN’ motif is shown as magenta spheres, and the ‘HR’ motif is shown as light brown spheres. The RNA template Le10 is colored yellow. The viral EBOV L-VP35-RNA complex is colored pink.

During the final review process of our manuscript, the cryo-EM structure of viral RNA promoter-bound EBOV polymerase (L–VP35-RNA) complex was published^33^. Comparison of the EBOV L–VP35-RNA (PDB: 8JSM) and our Le10-bound RSV polymerase (PDB: 8SNX) shows that they share a similar overall structure (Extended Data Fig. 8a). However, in the structure of the EBOV polymerase complex, the first 3 nt of the RNA are located in the catalytic pocket and have no interactions with the supporting loop or helix (Extended Data Fig. 8b). Another difference lies in the gate region (T8–T10 of RSV Le10; T7–T10 of EBOV RNA) of the template entrance channel, which involved three structural differences in L proteins: residues 220–233 of RSV L corresponding to residues 158–173 of EBOV L; residues 554–581 of RSV L corresponding to residues 499–515 of EBOV L; residues 1144–1158 of RSV L corresponding to residues 1051–1069 of EBOV L (Extended Data Fig. 8c).

**Extended Data Fig. 9:**
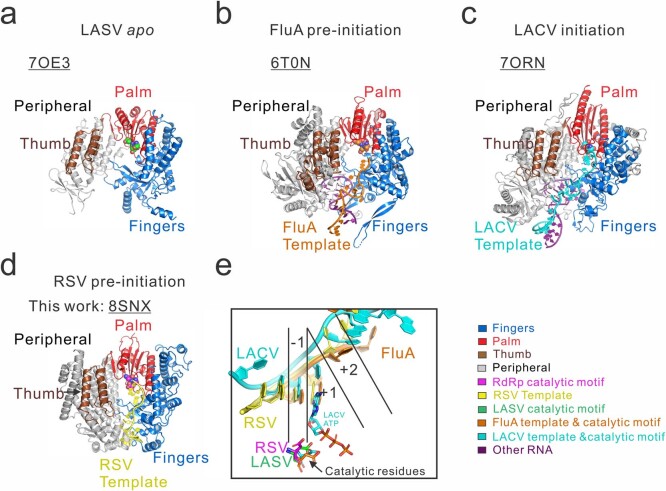
Structural comparison between the RSV RNA polymerase and RNA polymerases from viruses other than NNS  RNA viruses. (**a**) The RdRp domain of Lassa virus (LASV) *apo* polymerase (PDB: 7OE3). (**b**) The RdRp domain and RNA of Bat Influenza A (FluA) polymerase pre-initiation complex (PDB: 6T0N). (**c**) The RdRp domain and RNA of La Crosse virus (LACV) polymerase at initiation stage (PDB: 7ORN). (**d**) The RdRp domain and RNA of RSV polymerase at pre-initiation state (PDB: 8SNX, this work). (**e**) The comparison of the RNAs and catalytic residues among the polymerases from (**a**-**d**). The catalytic sites −1, +1, and +2 are labeled. An ATP is located at the +1 catalytic site in the LACV polymerase initiation complex. The RdRp domains of the RNA polymerase were shown as conventional “fingers-palm-thumb” right-hand motifs, which are displayed as the fingers (blue), the palm (red), and the thumb (brown). The PDB accession codes are underlined.

We also compared the RNA polymerases between RSV and viruses other than NNS RNA viruses, such as Lassa virus (LASV) apo polymerase (PDB: 7OE3)^34^, bat influenza A (FluA) pre-initiation complex (PDB: 6T0N)^35^ and La Crosse virus (LACV) initiation complex (PDB: 7ORN)^36^, in Extended Data Fig. 9. The RdRp domains of these viruses’ polymerases all adopt conventional ‘fingers–palm–thumb’ right-hand motifs (Extended Data Fig. 9a–d) and the RNA templates share similar conformation in the catalytic sites −1 to +2 (Extended Data Fig. 9e). Additionally, we noticed that the RNA templates in these polymerases all have a twist between the catalytic positions +1 and +2, and the twist angle is the biggest in RSV pre-initiation complex (Extended Data Fig. 9e).

To better understand the mechanism of RNA synthesis initiation of RSV polymerase, we modelled the first 2 nt of the RNA product at the catalytic site −1 and +1 between the template and the catalytic ‘GDN’ motif (Fig. 4c, modelled RNA product). Comparing RSV polymerase with those of VSV and RABV, we observed that the side-chain indole groups of W1167 in the VSV L priming loop and W1180 in the RABV L priming loop are parallel to the base of the first nucleotide of the RSV RNA product at the catalytic site −1 (Fig. 4c). W1167 (VSV) and W1180 (RABV) have been proposed to play a critical role and serve as the priming residue for de novo RNA synthesis initiation, as they engage in a π–π interaction with the base of the first nucleotide^7–9,23^. However, we also noticed that the intrusion loops from PIV5 and NDV are distant from the modelled RNA product (Fig. 4c).

## Discussion

RSV is a significant cause of respiratory infections worldwide. The RSV polymerase, composed of the L and P proteins, has a vital role in RSV RNA synthesis. The RSV polymerase catalyses RdRP RNA synthesis, which consists of the initiation, elongation and termination stages. Understanding the atomic interactions between the RSV polymerase and its RNA template during RNA synthesis is crucial to developing effective treatments and preventative measures against RSV. Although the structures of the apo RNA polymerases from several NNS RNA viruses show overall similar architecture, except for the varying locations of the priming loop and the visibility of the supporting helix, the structures of RNA-bound polymerase have not yet been reported.

To fill the knowledge gap, we determined two of the first cryo-EM structures of an NNS viral polymerase in complex with its RNA promoters, shedding light on the mechanisms of RNA synthesis in NNS RNA viruses. Our studies revealed that binding of the RNA promoters to the polymerase leads to specific conformational changes, including tighter packing of the RdRp and Cap domains to create a smaller catalytic pocket and stabilization of the supporting helix. We also found that the template RNA enters the catalytic pocket from the bottom of the bowl formed by the Cap and RdRp domains to initiate RNA synthesis, and the RNA template was speculated to exit through the centre of the Cap domain during RNA synthesis^9,12^.

**Fig. 5:**
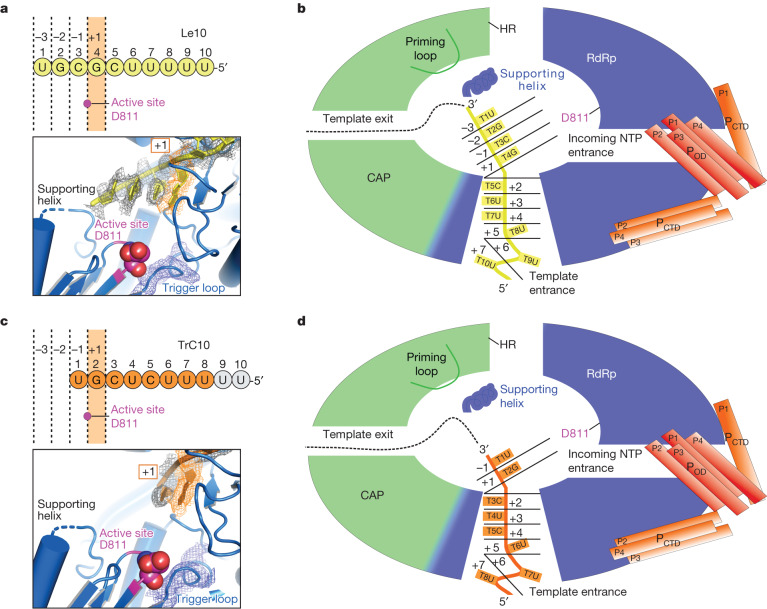
The models of the RSV polymerase (L–P) at the position 3 and 1 pre-initiation states. **a**–**d**, The RSV polymerase in complex with Le10, representing the position 3 pre-initiation state (**a**,**b**), and TrC10, representing the position 1 pre-initiation state (**c**,**d**). **a**,**c**, The sequence positions (from the 3′ end, 1−10) and catalytic positions (+1, −1, −2 and −3) of the RNA are shown above the actual residues in yellow (Le10) and orange (TrC10) circles. The active site D811 is shown as a magenta dot. The bottom panels show the D811 (magenta sphere) and the density map (mesh) of the RNA templates Le10 (at the counter level of 1.5*σ*) and TrC10 (at the counter level of 2.5*σ*) and trigger loops (at the counter level of 3.5*σ*). **b**,**d**, Schematic models of the RSV polymerase in complex with the Le10 (**b**) and TrC10 (**d**) promoters. Domains are coloured as in Fig. 1.

**Extended Data Fig. 10:**
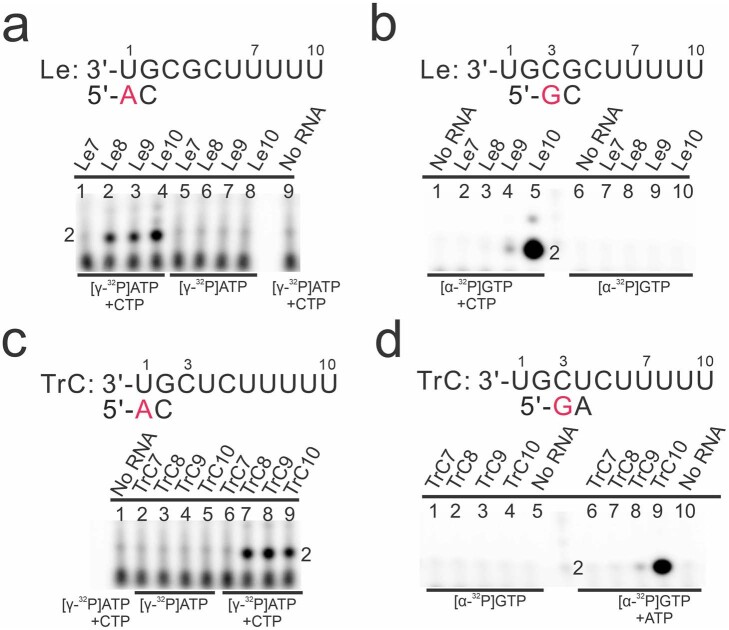
RSV RNA synthesis initiation at positions 1 and 3 testing with Leader (Le) or trailer complementary (TrC) promoters. (**a**) RSV RNA synthesis initiation at position 1 of Le10 labeled with [γ−^32^P]-ATP. (**b**) RSV RNA synthesis initiation at position 3 of Le10 labeled with [α−^32^P]-GTP. (**c**) RSV RNA synthesis initiation at position 1 of TrC10 labeled with [γ−^32^P]-ATP. (**d**) RSV RNA synthesis initiation at position 3 of TrC10 labeled with [α−^32^P]-GTP. Corresponding NTPs used for each sample are labeled below the figure. ATP at 50 μM with 5 μCi of [γ−^32^P] ATP or CTP at 1.25 mM and ATP at 50 μM with 5 μCi of [γ−^32^P] ATP were used in panels (**a**) and (**c**); GTP at 50 μM with 5 μCi of [α−^32^P] GTP or CTP at 1.25 mM and GTP at 50 μM with 5 μCi of [α−^32^P] GTP were used in panel (**b**); GTP at 50 μM with 5 μCi of [α−^32^P] GTP or ATP at 1.25 mM and GTP at 50 μM with 5 μCi of [α−^32^P] GTP were used in panel (**d**). Reactions without RNA templates with corresponding NTPs were used as controls. Position 1 required a minimum template length of 8 nts for initiation, whereas position 3 required a minimum length of 10 nts. All data shown are representative of three independent experiments (n  =  3), and the uncropped autoradiograph images are shown in Supplementary Fig. 1.

Our studies explain the unique ability of RSV polymerase to initiate RNA synthesis at either position 1 or 3 of the RNA promoters from the genome and antigenome^19–23^. Our structural models of the polymerase in complex with 10-nt promoters from Le and TrC regions show different pre-initiation positions. We reveal that the polymerase prefers to initiate RNA synthesis at positions 3 of the Le10 promoter and 1 of the TrC10 promoter (Fig. 5a,c). The in vitro RSV RNA synthesis assay revealed that for both the Le and TrC promoters, a minimum 8-nt template length is required for position 1 initiation, and a 10-nt template length is needed for position 3 initiation^24^ (Extended Data Fig. 10). Although the Le10-bound complex has two more nucleotides in the catalytic pocket than the TrC-bound complex, they share 8 nt—of which 6 nt are in the template entrance channel, and 2 nt are in the catalytic pocket (Fig. 5b,d). This finding explains the minimum template length requirements observed in the assay^24^.

The interactions between the RNA templates and the RSV polymerase contribute to the formation of the pre-initiation complex. This study demonstrated that positions 3 and 4 of Le10 and 1 and 2 of TrC10 occupy the −1 and +1 catalytic sites, respectively. The catalytic residue D811 from the RdRp domain is opposite to the −1 and +1 sites (Fig. 5). In the structures of Le10- and TrC10-bound RSV polymerase, the +1 catalytic sites are both occupied by G. We observed interactions between the G base and residues of the surrounding RdRp, such as K619, F629 and E778 (Figs. 2b and 3b,c(II)), which are strictly conserved in the RdRp domains of NNS viral polymerases (Extended Data Fig. 5a). Our findings suggest that the initiation process of other NNS RNA viruses may be similar to that of RSV. Positions 5–10 of Le10 and 3–8 of TrC10 are situated in the template entrance channel and interact similarly with RSV L, which are all pyrimidines (U or C), indicating smaller bases are preferred in the template entrance channel (catalytic cites +2 to +7; Fig. 5) during the formation of the pre-initiation complex. Our previous research showed that the RNA synthesis activity of the RSV polymerase is higher when positions 5–7 of the template TrC10 are pyrimidines (U or C) rather than purines (A or G)^24^. In addition, we observed that the RNA residues twist at the catalytic sites +2 and +5 in both promoter-bound structures (Fig. 5b,d). In the Le10-bound structure, there are twists at positions 5 and 8, followed by a 90° rotation for the bases of positions 8–10 (Fig. 5b). The twists occur at positions 3 and 6 in the TrC10-bound polymerase structure (Fig. 5d). We speculate that the twist may also favour a small pyrimidine base of U or C at the twist pivot point during RNA synthesis initiation, which explains why positions 3, 5 and 8 of the Le and TrC templates were found to be critical in a previous in vitro RSV RNA synthesis assay^18,24^. The twisted structure of the RNA template in the template entrance channel may also help to stabilize the RNA conformation, such as the G in the +1 catalytic site, in the pre-initiation state.

The cryo-EM structures of the RSV polymerase complexed with the viral RNA promoters presented in this study provide a better understanding of the RNA synthesis initiation process. To our knowledge, these structures are two of the first structures of an NNS viral polymerase in complex with its RNA templates to have been determined. As the polymerases from NNS RNA viruses are similar, nearly all containing a supporting helix with a supporting loop, our findings allow us to extrapolate the interactions of conserved residues to other NNS viral polymerases. Understanding the complex enzymatic function of RSV RNA synthesis is essential for developing effective antiviral drugs. These findings provide a mechanistic understanding of the RNA synthesis process, which will be valuable in developing effective treatments and preventative measures against RSV and other NNS RNA viruses.

## Methods

### Expression and purification of the RSV polymerase (L–P complex)

The expression and purification of the RSV polymerase (L–P complex) were carried out as follows. The codon-optimized helper plasmids of the RSV (strain A2) L and P proteins were obtained from BEI Resources. The L and P genes were subcloned into the pFastBac Dual vector (Invitrogen) with the RSV L gene at open reading frame 1 and the RSV P gene at open reading frame 2. A 6×His tag was added to the N terminus of the RSV L protein, separated by a TEV protease cleavage site. Then, the recombinant pFastBac Dual vector was transformed into *Escherichia coli* DH10Bac for bacmid DNA generation. The Cellfectin II reagent (Thermo Fisher Scientific) was used to transfect the bacmid DNA into Sf21 cells (Thermo Fisher Scientific) to obtain the recombinant baculoviruses. Sf21 cells were infected by the recombinant baculoviruses in suspension culture and collected 72 h post-infection by centrifugation for 15 min at 1,000*g*. The collected cells were resuspended in lysis buffer (50 mM sodium phosphate pH 7.4, 300 mM NaCl, 6 mM MgSO_4_, 10% glycerol, 0.2% NP-40, EDTA-free protease inhibitor), lysed with a homogenizer and clarified through centrifugation for 60 min at 16,000*g*. The clarified lysate was incubated with Co^2+^-NTA agarose resin (GoldBio) and washed with wash buffer (50 mM sodium phosphate pH 7.4, 300 mM NaCl, 6 mM MgSO_4_, 10% glycerol, 10 mM imidazole), and the RSV L–P complexes were eluted with elution buffer (50 mM sodium phosphate pH 7.4, 300 mM NaCl, 6 mM MgSO_4_, 10% glycerol, 250 mM imidazole). The eluted sample was treated with TEV enzyme and applied to Co^2+^-NTA agarose resin. The flowthrough sample was applied to a heparin column and further purified by size-exclusion chromatography with gel filtration buffer (25 mM HEPES pH 7.4, 300 mM NaCl, 6 mM MgSO_4_, 0.5 mM tris(2-carboxyethyl) phosphine hydrochloride (TCEP)) using a Superose 6 Increase 10/300 GL column (GE Healthcare). SDS–PAGE was used to analyse the quality of purified proteins. The pure proteins were stored in 30-μl aliquots at −80 °C after being flash-frozen in liquid nitrogen for further use. These procedures have been described previously^10^.

### In vitro RNA synthesis assay

In the RNA synthesis assay, RNA promoter sequences with different lengths of the Le region of the genome and TrC region of the antigenome were used. The oligonucleotides, such as Le10 and TrC10, were chemically synthesized by Integrated DNA Technologies (Coralville, IA, USA) or Horizon Discovery (Waterbeach, UK) and had hydroxyl (OH) groups at both 3′ and 5′ terminals.

Radioactive isotope-labelled nucleotides, [α-^32^P]GTP and [γ-^32^P]ATP, were purchased from Perkin Elmer. The reaction mixtures consisted of 2 μM RNA template (without RNA template as the control), the RSV L–P complexes (about 300 ng RSV L), NTPs (ATP at 50 μM with 5 μCi of [γ-^32^P]ATP or CTP at 1.25 mM and ATP at 50 μM with 5 μCi of [γ-^32^P]ATP were used in Extended Data Fig. 10a,c; GTP at 50 μM with 5 μCi of [α-^32^P]GTP or CTP at 1.25 mM and GTP at 50 μM with 5 μCi of [α-^32^P]GTP were used in Extended Data Fig. 10b; GTP at 50 μM with 5 μCi of [α-^32^P]GTP or ATP at 1.25 mM and GTP at 50 μM with 5 μCi of [α-^32^P]GTP were used in Extended Data Fig. 10d) and reaction buffer (50 mM Tris-HCl pH 7.4, 8 mM MgCl_2_, 5 mM dithiothreitol, 10% glycerol) in a final volume of 20 μl. The reaction mixtures were incubated at 30 °C for 2 h and heated to 90 °C for 5 min. Then, 5 μl of the stop buffer (90% formamide, 20 mM EDTA, 0.02% bromophenol blue) was added to each reaction mixture (Extended Data Fig. 10). The isotope-labelled nucleotides with the same concentration were purchased fresh and used for the reactions. Only the reaction mixtures containing the same radioactive isotope-labelled NTPs were directly compared for clarity. The RNA products were analysed by electrophoresis on a 20% polyacrylamide gel containing 7 M urea in a Tris–borate–EDTA buffer, followed by phosphorimaging with a Typhoon FLA 7000 scanner (GE Healthcare). The molecular weight ladders were generated by labelling Tr5, Tr7, Tr14, Tr21 and Tr25 with [γ-^32^P]ATP using T4 polynucleotide kinase (M0201L, NEB) following the protocols of the manufacturer (NEB).

### Cryo-EM grid specimen preparation and data acquisition

We incubated 0.35 mg ml^−1^ purified RSV polymerase with 30 µM Le10, 150 µM GTP and CpNpp or 30 µM TrC10, 150 µM GTP and ApNpp at room temperature for 1 h. Subsequently, we applied 3.0 μl of the assembled complexes onto glow-discharged UltrAuFoil 300 mesh R1.2/1.3 grids (Electron Microscopy Sciences) for Le10 and TrC10, respectively. After this, we blotted the grids for 3 s at about 100% humidity and flash-froze them in liquid ethane using an FEI Vitrobot Mark ΙV.

Images were collected with Leginon 3.5 on FEI Titan Krios microscopes operated at an acceleration voltage of 300 kV with a Gatan K3 camera with a 1.058 Å pixel size. The defocus range was set from −0.8 μm to −2.5 μm. Dose-fractionated images were recorded with a per-frame exposure time of 2,000 ms and a dose of about 1.305 electrons per square ångström per frame. The total accumulated dose was about 52.21 electrons per square ångström. A total of 6,741 micrographs were collected for the Le10-bound RSV polymerase, and 7,777 micrographs were collected for the TrC10-bound RSV polymerase.

### Cryo-EM data processing

Motion correction of the data for RSV polymerase in complex with Le10 was carried out with the program MotionCor2 (ref. ^37^). The contrast transfer function was estimated using the program CTFFIND4 (ref. ^38^). A total of 3,658,410 particles were auto-picked by crYOLO^27^, and a box size of 200 pixels was used to extract the particles. Particle 2D classification, initial 3D model building, 3D classification, 3D refinement, contrast transfer function refinement and polishing were carried out using RELION 3.1.3 (ref. ^26^). The final refinement was validated using cisTEM^39^, using the best class as the initial model. The global search was carried out once without the mask, followed by another global search using a soft mask (6-pixel soft edge) generated in RELION. After 2D and 3D classifications, 358,385 particles were selected for final 3D refinement and polishing, resulting in a cryo-EM map with a resolution of 3.40 Å. The TrC10-bound RSV polymerase dataset was processed using a similar method. A total of 3,646,076 particles were picked for further data processing. Finally, 197,859 particles were selected after 2D and 3D classifications and subjected to final 3D refinement and polishing, yielding a cryo-EM map with a resolution of 3.41 Å.

All reported resolutions were based on gold-standard refinement procedures and the Fourier shell correlation = 0.143 criterion. The local resolution was estimated using ResMap^40^. Further data processing and refinement details are summarized in Extended Data Figs. 1 and 2 and Supplementary Table 1.

### Model building and figure preparation

The initial model docked into the cryo-EM map for the RSV polymerase complex with Le10 or TrC10 was the apo RSV polymerase coordinates (PDB: 6UEN). UCSF Chimera and COOT were used for fitting the initial model^28,29^. The final structures of the RSV polymerase in complex with Le10 or TrC10 were built and refined using COOT and PHENIX, and the model geometries were validated using MolProbity^29–31^. Supplementary Table 1 summarizes the data collection and model refinement statistics. The software used in this project was curated by SBGrid^41^. All of the figures representing model and electron density maps were generated using COOT^29^, UCSF Chimera^25^ and PyMOL^32^. Multiple sequence alignment was carried out using Multalin^42^ and ESPript^43^.

### Reporting summary

Further information on research design is available in the Nature Portfolio Reporting Summary linked to this article.

## Online content

Any methods, additional references, Nature Portfolio reporting summaries, source data, extended data, supplementary information, acknowledgements, peer review information; details of author contributions and competing interests; and statements of data and code availability are available at 10.1038/s41586-023-06867-y.

### Supplementary information

Supplementary Information Supplementary Fig. 1 and Table 1. Supplementary Fig. 1: Uncropped SDS–PAGE gels and autoradiographs used to prepare Fig. 1b and Extended Data Fig. 10. Supplementary Table 1: Cryo-EM data collection, refinement and validation statistics.

Reporting Summary

## Data Availability

The cryo-EM density maps and atomic coordinates have been deposited to the Electron Microscopy Data Bank (EMDB; https://www.ebi.ac.uk/emdb/) and the PDB (https://www.rcsb.org), respectively, with the following accession numbers: Le10-bound RSV polymerase (L–P) complex (EMD-40641, PDB 8SNX) and TrC10-bound RSV polymerase (L–P) complex (EMD-40642, PDB 8SNY).
