# Supplementary material for: Structures of the promoter-bound respiratory syncytial virus polymerase
Source: Nature. 2023 Dec 20;625(7995):611–7. doi: 10.1038/s41586-023-06867-y (PMC10794133; doi:10.1038/s41586-023-06867-y)
Supplement: Supplementary file 1 — Supplementary Fig. 1 and Table 1. Supplementary Fig. 1: Uncropped SDS–PAGE gels and autoradiographs used to prepare Fig. 1b and Extended Data Fig. 10. Supplementary Table 1: Cryo-EM data collection, refinement and validation statistics. [file 41586_2023_6867_MOESM1_ESM.docx]

**Supplementary information**

**Structures of the promoter-bound respiratory syncytial virus polymerase**

Dongdong Cao^1^, Yunrong Gao^1^, Zhenhang Chen^1^, Inesh Gooneratne^1^, Claire Roesler^1^, Cristopher Mera^1^, Paul D'Cunha^1^, Anna Antonova^1^, Deepak Katta^1^, Sarah Romanelli^1^, Qi Wang^1^, Samantha Rice^1^, Wesley Lemons^1^, Anita Ramanathan^1^, Bo Liang^1*^

^1^Department of Biochemistry, Emory University School of Medicine, Atlanta, GA, 30322 United States

^*^Correspondence: [bo.liang@emory.edu](mailto:bo.liang@emory.edu)

**Supplementary information**

Table of content:

Supplementary Figure 1. Uncropped SDS-PAGE gels, and autoradiographs used for preparing Fig. 1b, and Extended Data Fig. 10.

Supplementary Table 1. Cryo-EM data collection, refinement, and validation statistics.


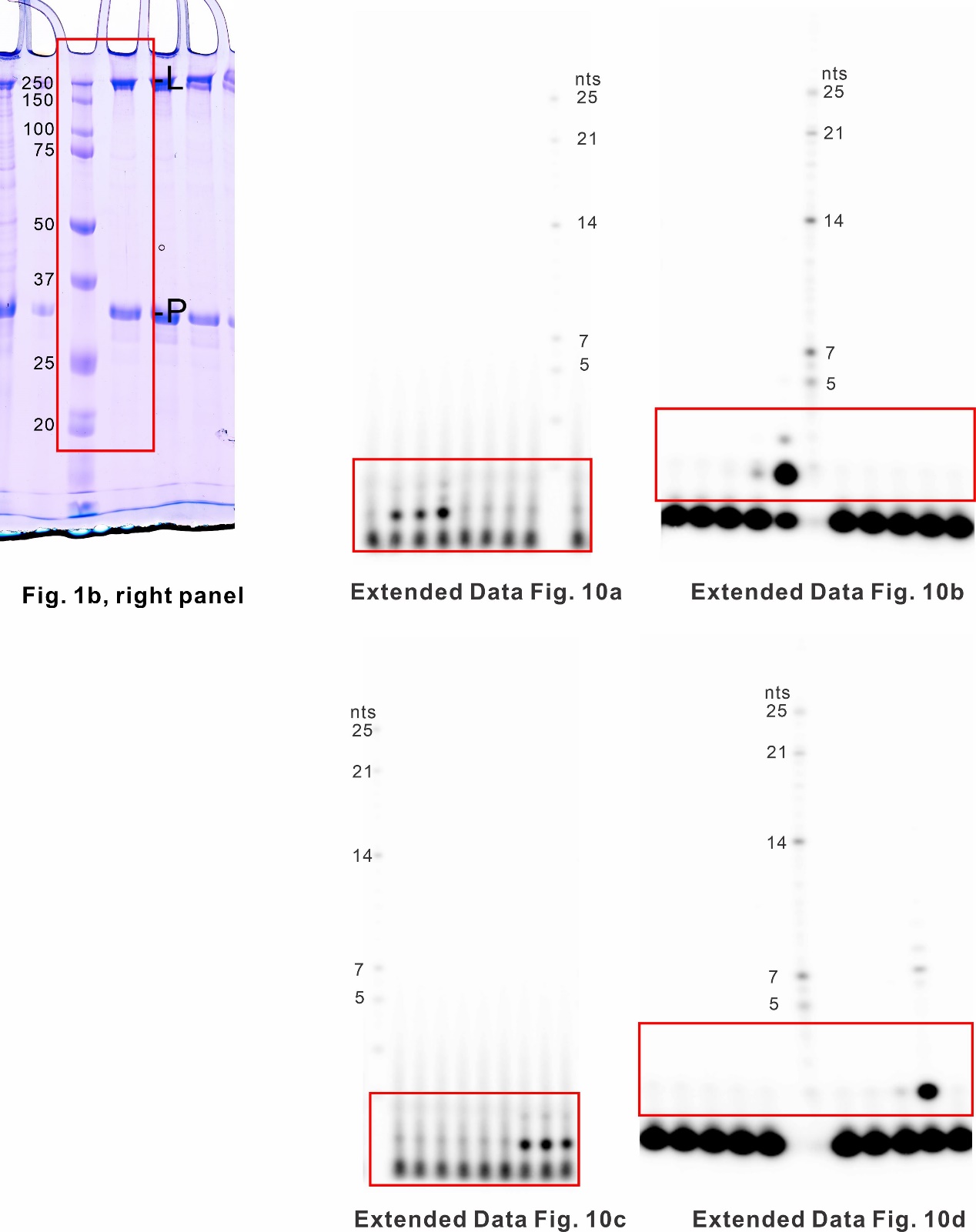


**Supplementary Figure 1**. Uncropped SDS-PAGE gels, and autoradiographs used for preparing Fig. 1b, and Extended Data Fig. 10. Red boxes indicate the cropped regions shown in the corresponding figures.

**Supplementary Table 1. Cryo-EM data collection, refinement, and validation statistics.**

|  | Le10-bound  RSV polymerase (L:P) | TrC10-bound  RSV polymerase (L:P) |
| --- | --- | --- |
| **PDB code** | 8SNX | 8SNY |
| **EMDB code** | EMD-40641 | EMD-40642 |
| **Data Collection and Processing** |  |  |
| Magnification | 81000 | 81000 |
| Voltage (kV) | 300 | 300 |
| Electron Exposure (e-/Å^2^) | 51.11 | 56.86 |
| Defocus range (μm) | -0.8 to -2.5 | -0.8 to -2.5 |
| Pixel size (Å) | 1.058 | 1.058 |
| Symmetry imposed | C1 | C1 |
| Initial particle images (no.) | 3,658,410 | 3,646,076 |
| Final particle images (no.) | 358,385 | 197,859 |
| Map resolution (Å) | 3.40 | 3.41 |
| FSC threshold | 0.143 | 0.143 |
|  |  |  |
| **Refinement** |  |  |
| Initial model used (PDB code) | 6UEN | 6UEN |
| Map sharpening *B* factor (Å^2^) | -155.5 | -146.1 |
| Model composition |  |  |
| Non-hydrogen atoms | 13,741 | 13,744 |
| Protein residues | 1675 | 1680 |
| Nucleotide | 10 | 8 |
| *B* factors (Å^2^) |  |  |
| Protein | 53.07 | 56.84 |
| Nucleotide | 58.69 | 65.02 |
| R.M.S. deviations |  |  |
| Bond lengths (Å) | 0.003 | 0.004 |
| Bond angles (°) | 0.566 | 0.625 |
| Validation |  |  |
| MolProbity score | 1.70 | 1.87 |
| Clashscore | 10.13 | 13.27 |
| Poor rotamers (%) | 0.33 | 0.33 |
| Ramachandran statistics |  |  |
| Favored (%) | 96.98 | 96.45 |
| Allowed (%) | 3.02 | 3.55 |
| Disallowed (%) | 0 | 0 |
