# Supplementary material for: Structures of the promoter-bound respiratory syncytial virus polymerase
Source: Nature. 2023 Dec 20;625(7995):611–7. doi: 10.1038/s41586-023-06867-y (PMC10794133; doi:10.1038/s41586-023-06867-y)
Supplement: Supplementary file 2 — Reporting Summary [file 41586_2023_6867_MOESM2_ESM.pdf]

nature portfolio

Corresponding author(s): Bo Liang

Last updated by author(s): Oct 21, 2023

Reporting Summary

Nature Portfolio wishes to improve the reproducibility of the work that we publish. This form provides structure for consistency and transparency in reporting. For further information on Nature Portfolio policies, see our Editorial Policies and the Editorial Policy Checklist.

Statistics

For all statistical analyses, confirm that the following items are present in the figure legend, table legend, main text, or Methods section.

n/a	Confirmed	
☐	☒	The exact sample size (*n*) for each experimental group/condition, given as a discrete number and unit of measurement
☐	☒	A statement on whether measurements were taken from distinct samples or whether the same sample was measured repeatedly
☒	☐	The statistical test(s) used AND whether they are one- or two-sided<br>*Only common tests should be described solely by name; describe more complex techniques in the Methods section.*
☒	☐	A description of all covariates tested
☒	☐	A description of any assumptions or corrections, such as tests of normality and adjustment for multiple comparisons
☒	☐	A full description of the statistical parameters including central tendency (e.g. means) or other basic estimates (e.g. regression coefficient) AND variation (e.g. standard deviation) or associated estimates of uncertainty (e.g. confidence intervals)
☒	☐	For null hypothesis testing, the test statistic (e.g. *F*, *t*, *r*) with confidence intervals, effect sizes, degrees of freedom and *P* value noted<br>*Give P values as exact values whenever suitable.*
☒	☐	For Bayesian analysis, information on the choice of priors and Markov chain Monte Carlo settings
☒	☐	For hierarchical and complex designs, identification of the appropriate level for tests and full reporting of outcomes
☒	☐	Estimates of effect sizes (e.g. Cohen's *d*, Pearson's *r*), indicating how they were calculated

Our web collection on statistics for biologists contains articles on many of the points above.

Software and code

Policy information about availability of computer code

Data collection	Leginon 3.5
Data analysis	RELION v3.1.3, MotionCor2, CTFFIND4, crYOLO v1.7.6, cisTEM v1.0.0-beta, UCSF ChimeraX v1.6.1, COOT v0.9.8.1, PHENIX v1.19.2, ResMap v1.95, PyMOL v2.5.5, Molprobity v4.5.2

For manuscripts utilizing custom algorithms or software that are central to the research but not yet described in published literature, software must be made available to editors and reviewers. We strongly encourage code deposition in a community repository (e.g. GitHub). See the Nature Portfolio guidelines for submitting code & software for further information.

Data

Policy information about availability of data

All manuscripts must include a data availability statement. This statement should provide the following information, where applicable:

- Accession codes, unique identifiers, or web links for publicly available datasets
- A description of any restrictions on data availability
- For clinical datasets or third party data, please ensure that the statement adheres to our policy

The cryo-EM density maps and atomic coordinates have been deposited to the Electron Microscopy Data Bank (https://www.ebi.ac.uk/emdb/) and the Protein Data Bank (https://www.rcsb.org), respectively. The accession numbers are as follows: Le10-bound RSV polymerase (L:P) complex (EMD-40641, PDB 8SNX) and TrC10-bound RSV polymerase (L:P) complex (EMD-40642, PDB 8SNY).

Research involving human participants, their data, or biological material

Policy information about studies with human participants or human data. See also policy information about sex, gender (identity/presentation), and sexual orientation and race, ethnicity and racism.

Reporting on sex and gender	Not applicable
Reporting on race, ethnicity, or other socially relevant groupings	Not applicable
Population characteristics	Not applicable
Recruitment	Not applicable
Ethics oversight	Not applicable

Note that full information on the approval of the study protocol must also be provided in the manuscript.

Field-specific reporting

Please select the one below that is the best fit for your research. If you are not sure, read the appropriate sections before making your selection.

☒ Life sciences ☐ Behavioural & social sciences ☐ Ecological, evolutionary & environmental sciences

For a reference copy of the document with all sections, see nature.com/documents/nr-reporting-summary-flat.pdf

Life sciences study design

All studies must disclose on these points even when the disclosure is negative.

Sample size	No statistical methods were used to predetermine the sample size. The sample size of the cryo-EM data was chosen based on the sufficient number of particles to achieve high-resolution reconstructions. We also processed the data in parallel with the data collection to make sure the sample size is large enough to achieve high-resolution reconstructions. A total of 6,741 micrographs were collected for the Le10-bound RSV polymerase, and 7,777 micrographs were collected for the TrC10-bound RSV polymerase. Both provided sufficient particles to achieve high resolution structures.
Data exclusions	No data were excluded.
Replication	The in vitro RNA synthesis assay was repeated twice to confirm the results. All attempts at replication were successful.
Randomization	Randomization is not relevant to this study as no clinical trails or drug treatment assays were performed and no grouping was needed.
Blinding	Blinding is not relevant to this study as no clinical trails or drug treatment assays were performed and no grouping was needed for this study.

Reporting for specific materials, systems and methods

We require information from authors about some types of materials, experimental systems and methods used in many studies. Here, indicate whether each material, system or method listed is relevant to your study. If you are not sure if a list item applies to your research, read the appropriate section before selecting a response.

Materials & experimental systems

n/a	Involved in the study	
☒	☐	Antibodies
☐	☒	Eukaryotic cell lines
☒	☐	Palaeontology and archaeology
☒	☐	Animals and other organisms
☒	☐	Clinical data
☒	☐	Dual use research of concern
☒	☐	Plants

Methods

n/a	Involved in the study	
☒	☐	ChIP-seq
☒	☐	Flow cytometry
☒	☐	MRI-based neuroimaging

Eukaryotic cell lines

Policy information about cell lines and Sex and Gender in Research

Cell line source(s)	Sf21 cells derived from the parental Spodoptera frugiperda cell line IPLB-Sf-21-AE are purchased from thermofisher scientific.
Authentication	Sf21 cell line is insect cell line and the cell line used was not authenticated.
Mycoplasma contamination	This cell line was not tested for Mycoplasma contamination.
Commonly misidentified lines (See ICLAC register)	No commonly misidentified cell lines used in this study.
